# Genetic variations in regions of bovine and bovine-like enteroviral 5’UTR from cattle, Indian bison and goat feces

**DOI:** 10.1186/s12985-016-0468-8

**Published:** 2016-01-25

**Authors:** Nathamon Kosoltanapiwat, Marnoch Yindee, Irwin Fernandez Chavez, Pornsawan Leaungwutiwong, Poom Adisakwattana, Pratap Singhasivanon, Charin Thawornkuno, Narin Thippornchai, Amporn Rungruengkitkun, Juthamas Soontorn, Sasipan Pearsiriwuttipong

**Affiliations:** Department of Microbiology and Immunology, Faculty of Tropical Medicine, Mahidol University, Bangkok, Thailand; Faculty of Veterinary Science, Mahidol University, Bangkok, Thailand; Department of Tropical Hygiene, Faculty of Tropical Medicine, Mahidol University, Bangkok, Thailand; Department of Helminthology, Faculty of Tropical Medicine, Mahidol University, Bangkok, Thailand; Department of Molecular Tropical Medicine and Genetics, Faculty of Tropical Medicine, Mahidol University, Bangkok, Thailand

**Keywords:** Bovine enterovirus, Cattle, Goat, Indian bison, RT-PCR, 5’UTR, Thailand

## Abstract

**Background:**

Bovine enteroviruses (BEV) are members of the genus *Enterovirus* in the family *Picornaviridae*. They are predominantly isolated from cattle feces, but also are detected in feces of other animals, including goats and deer. These viruses are found in apparently healthy animals, as well as in animals with clinical signs and several studies reported recently suggest a potential role of BEV in causing disease in animals. In this study, we surveyed the presence of BEV in domestic and wild animals in Thailand, and assessed their genetic variability.

**Methods:**

Viral RNA was extracted from fecal samples of cattle, domestic goats, Indian bison (gaurs), and deer. The 5’ untranslated region (5’UTR) was amplified by nested reverse transcription-polymerase chain reaction (RT-PCR) with primers specific to BEV 5’UTR. PCR products were sequenced and analyzed phylogenetically using the neighbor-joining algorithm to observe genetic variations in regions of the bovine and bovine-like enteroviral 5’UTR found in this study.

**Results:**

BEV and BEV-like sequences were detected in the fecal samples of cattle (40/60, 67 %), gaurs (3/30, 10 %), and goats (11/46, 24 %). Phylogenetic analyses of the partial 5’UTR sequences indicated that different BEV variants (both EV-E and EV-F species) co-circulated in the domestic cattle, whereas the sequences from gaurs and goats clustered according to the animal species, suggesting that these viruses are host species-specific.

**Conclusions:**

Varieties of BEV and BEV-like 5’UTR sequences were detected in fecal samples from both domestic and wild animals. To our knowledge, this is the first report of the genetic variability of BEV in Thailand.

## Background

Bovine enteroviruses (BEV) are members of the genus *Enterovirus* in the family *Picornaviridae*. They are small (27–30 nm), non-enveloped, positive-stranded RNA viruses with an icosahedral virion and a genome of approximately 7.5 kb that contains a single long open reading frame (ORF) flanked by 5’ and 3’ untranslated regions (UTRs). The BEV genome encodes a single polypeptide, which is cleaved by viral proteases to produce viral proteins composed of structural proteins (VP1-VP4) and non-structural proteins involved in viral replication [1, 2].

The genus *Enterovirus* contains 12 species: enterovirus (EV) A, B, C, D, E, F, G, H, and J, and rhinovirus (RV) A, B, and C. BEV belong to species EV-E (formerly BEV-1 or BEV-A) and EV-F (formerly BEV-2 or BEV-B) [3, 4]. Comparison of their 5’UTR sequences can differentiate BEV from other enteroviruses. The 5’UTR is a relatively conserved genomic region that nevertheless varies between enteroviruses, making it useful for the detection and primary classification of the genus *Enterovirus* into groups, such as human, porcine, simian, and bovine enteroviruses [5–8]. The comparison of variable regions of this site is also useful for phylogenetic analyses [6, 9], and this is the sequence of choice for studying the enteroviruses. The enteroviral 5’UTR forms highly ordered secondary stem-loop structures composed of domains I, II, III, IV, V, and VI, and an additional domain VII in some enteroviruses, such as human, porcine, and simian enteroviruses [5, 9]. The cloverleaf structure at the very 5’ end (domain I) and the internal ribosome entry site (IRES) element (domains II-VI) are involved in viral plus-strand RNA synthesis and translation initiation, respectively [9]. In addition to the single cloverleaf structure found in the 5’UTR of all enteroviruses, the BEV 5’UTR contains two cloverleaf structures (domains I and I*), which are separated by a simple stem-loop structure (domain I**). This additional structure arises from an insertion of about 110 nucleotides in the area between the 5’ cloverleaf structure and the IRES region. Based on this typical 5’UTR characteristic, the BEV are classified phylogenetically as their own group in the genus *Enterovirus* [5]. BEV and other enteroviruses can be further classified into species, genotypes, or serotypes by molecular studies of capsid protein sequences, particularly VP1, VP2, and VP3 [5, 10, 11].

In various regions around the world, BEV have been predominantly isolated from cattle feces, but they have also been isolated from the feces of other animals, including sheep, goats, horses, geese, possums, and deer. [3, 5, 6, 12–14]. These viruses have been found in both healthy animals and animals with clinical signs of respiratory disease, enteric disease, or fertility disorders, and in the fetal fluids of aborted calves [5, 15, 16]. BEV are stable in the animal digestive tract and can be shed in a large quantity from apparently healthy animals [6, 12]. They can also persist in the environment for a long time and have been detected in samples from oysters and sewage water. Detection of the viruses is therefore useful as an indicator of environmental contamination by animal feces [6, 12, 17, 18]. Although it is believed that BEV are associated with clinical signs in cattle and calves, the role of these viruses in disease pathogenesis remains controversial. In previous studies, disease attributed to BEV could not be reproduced in experimental animals [16, 19]. However, in a more recent study, calves experimentally inoculated with the EV-E1 strain, while showing no clinical signs, had the virus localized within encephalitis and myocarditis lesions after acute infection [20]. Similarly, in experiments with suckling mice, inoculation with an isolated virus caused infection and intestinal, hepatic, and pulmonary pathologies [16]. The increased isolation of BEV from cattle with diarrhea and respiratory disease also indicates that BEV has the potential to cause disease and should be of concern to the animal husbandry industry [15].

Although BEV isolates from many countries have been characterized, including those from China, Japan, Pakistan, Australia, Germany, Spain, the United Kingdom, and the United States [2, 5, 6, 12, 14–16, 18], there have been no recent reports of the BEV infection status in Thailand, regarding either BEV epidemiology or genetic diversity. Therefore, the purpose of this study was to survey domestic and wild animals in areas of Kanchanaburi Province in western Thailand for BEV infection. Fecal samples from cattle, goats, Indian bison (gaurs), and deer were screened for the presence of BEV or BEV-like 5’UTR using nested reverse transcription (RT)-PCR. 5’UTR sequences retrieved from positive samples were analyzed phylogenetically to determine their genetic diversity.

## Results

### Detection of BEV 5’UTR

**Fig. 1:**
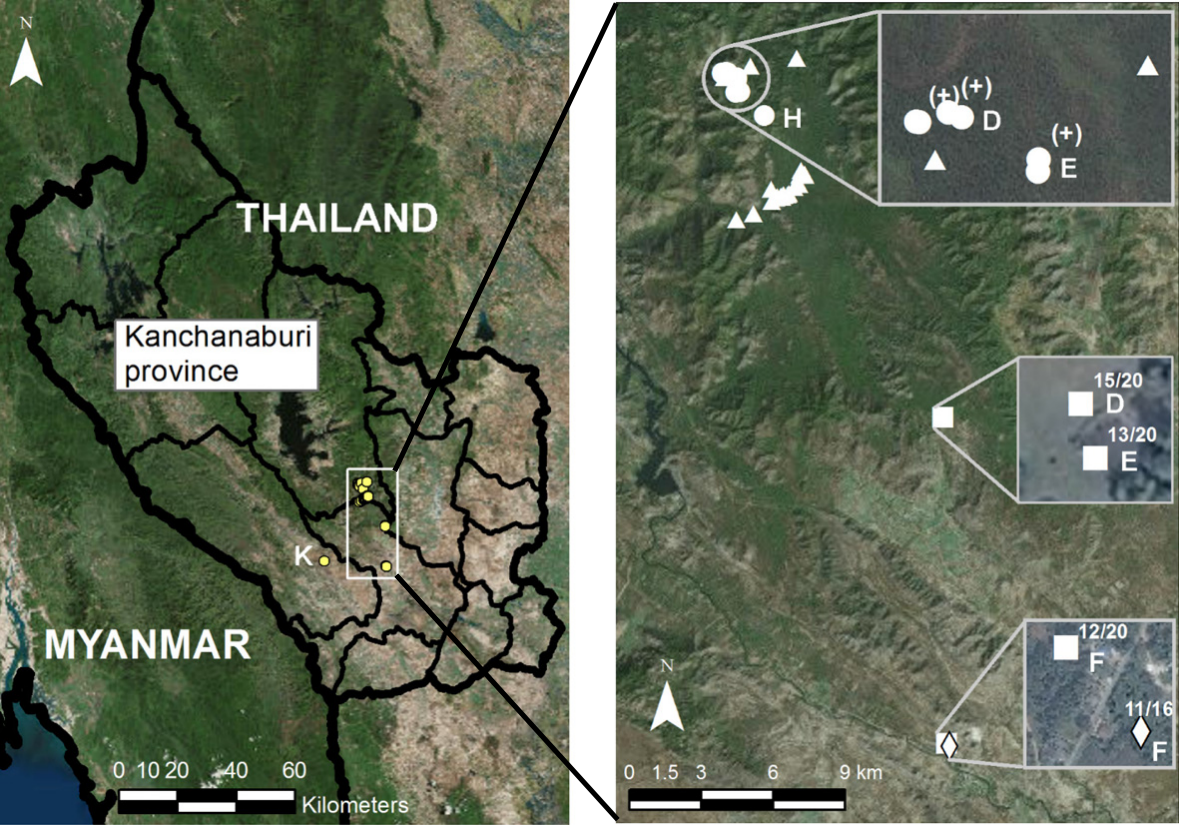
Specimen collection locations. (Left panel): The survey sites are located by yellow circlets. (Right panel): An enlargement of a rectangular area on the left panel is shown. Squares represent cattle samples, diamonds represent goat samples, circles represent gaur samples, and triangles represent deer samples. One symbol may represent more than one sample if collected in the same area. The numbers of positive samples from cattle and goats are indicated on the map, and (+) represents positive samples from gaurs. Sources: Esri, DigitalGlobe, GeoEye, Earthstar, Geographics, CNES/Airbus DS, USDA, USGS, AEX, Getmapping, Aerogrid, IGN, IGP, Swisstopo, and the GIS User Community

Partial nucleotide fragments of BEV and BEV-like 5’UTR (approximately 290 bp) were detected in fecal samples from domestic cattle (40/60, 67 %), wild gaurs (3/30, 10 %), and domestic goats (11/46, 24 %), but not in any of the deer samples tested in this study. The demographic data and the numbers of positive samples are shown in Table 1. The cattle samples were collected from three herds (groups 1, 2, and 3, corresponding to codes D, E, and F, respectively, in Table 1 and on the phylogenetic tree). All the cattle were from domestic herds that were released onto grassy fields to feed during the day and kept in barns overnight. The samples were collected from the barns. The goat samples were collected from two separate groups of domestic goats housed inside. The fecal samples from gaurs and deer were collected from forested land in different areas. The gaur samples were collected from both animals living separately (clans of 3–5 gaurs, designated groups 1 and 2, corresponding to codes D and E, respectively, in Table 1 and on the phylogenetic tree) and from animals living in a large group (group 3, code H). A map of the sample collection sites and the locations of samples positive for the viruses are shown in Fig. 1.

**Table 1:** Detection of BEV 5′UTR sequences in animal fecal samples

Animal	Group (code)	Collection date	Positive/total samples (%)	Total (%)
Cattle	1 (D)	Apr 2013	15/20 (75)	40/60 (67)
	2 (E)	May 2013	13/20 (65)	
	3 (F)	May 2013	12/20 (60)	
Domestic goat	1 (F)	May 2013	11/16 (69)	11/46 (24)
	2 (K)	Nov 2013	0/30 (0)	
Gaur	1 (D)	Apr 2013	2/4 (50)	3/30 (10)
	2 (E)	May 2013	1/5 (20)	
	3 (H)	Aug 2013	0/21 (0)	
Deer	-	Jan-May 2013	0/48 (0)	0/48 (0)

### 5’UTR sequence analysis

**Fig. 2:**
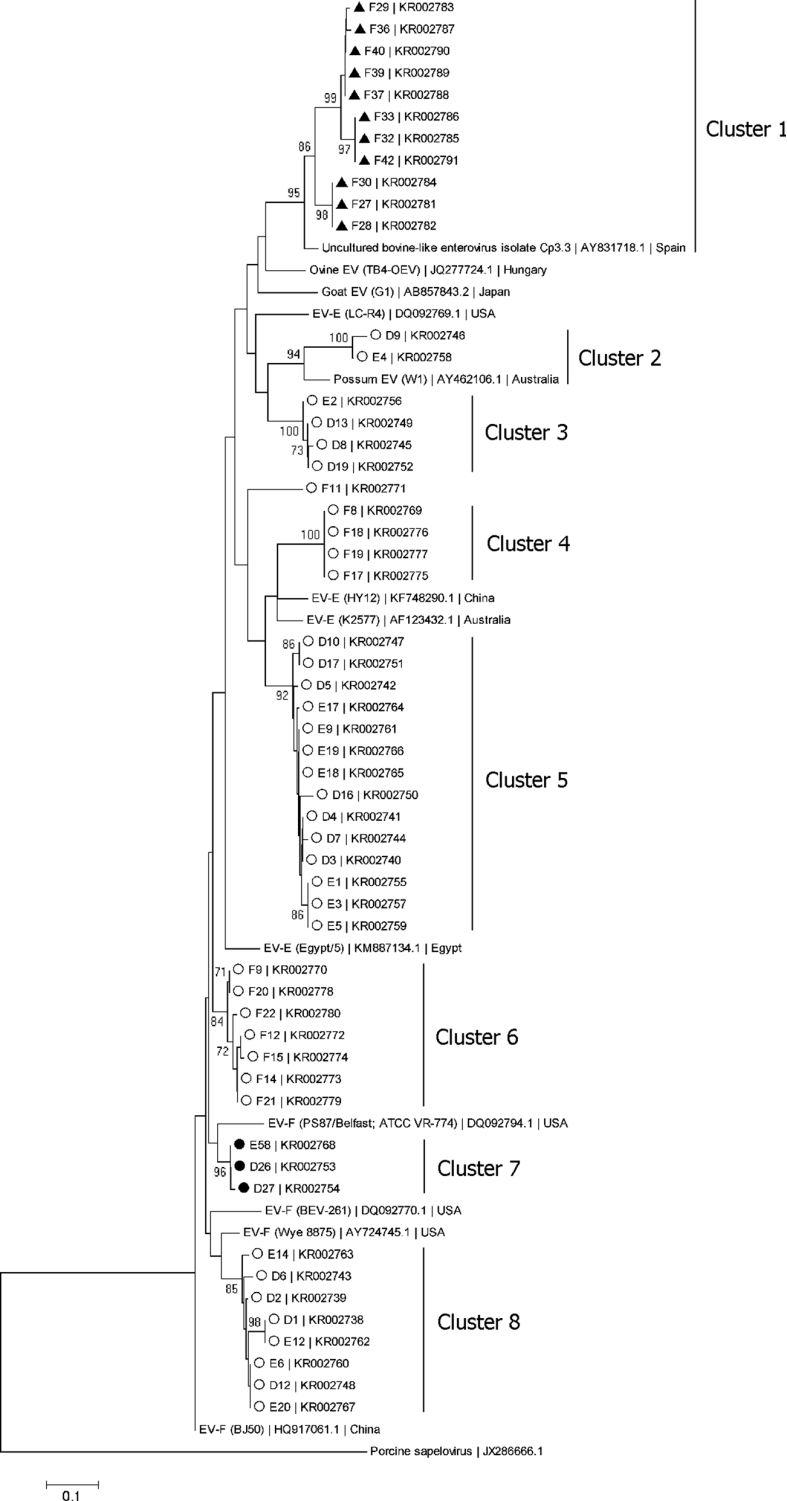
Phylogenetic analysis of 54 partial BEV and BEV-like 5′UTR sequences (158–162 nt). The phylogenetic tree was constructed with the neighbor-joining algorithm, the Kimura two-parameter distance model, and 1,000 bootstrap replicates. The isolate names and accession numbers of the nucleotide sequences determined in this study are shown. Open circles, closed circles, and closed triangles represent cattle, gaur, and goat samples, respectively. The isolate names (in parentheses), accession numbers, and countries of collection for the nucleotide sequences of other enteroviruses retrieved from the GenBank database are shown. The scale bar represents 0.1 nucleotide substitutions per site. Bootstrap values greater than 70 are indicated at the nodes. Porcine sapelovirus was used as the outgroup

**Fig. 3:**
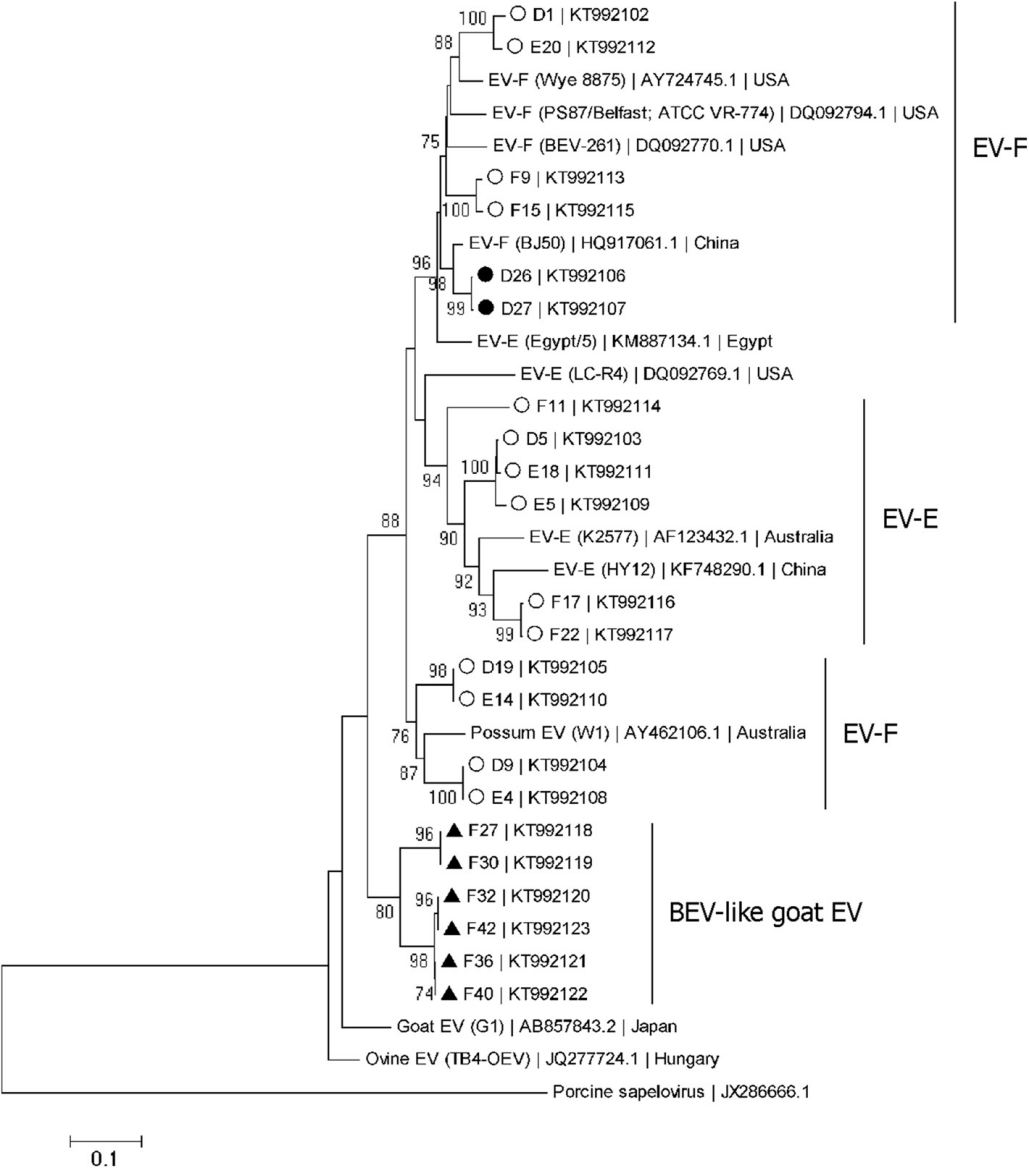
Phylogenetic analysis of 22 partial BEV and BEV-like 5′UTR sequences (546–556 nt). The phylogenetic tree was constructed with the neighbor-joining algorithm, the Kimura two-parameter distance model, and 1,000 bootstrap replicates. The isolate names and accession numbers of the nucleotide sequences determined in this study are shown. Open circles, closed circles, and closed triangles represent cattle, gaur, and goat samples, respectively. The isolate names (in parentheses), accession numbers, and countries of collection for the nucleotide sequences of other enteroviruses retrieved from the GenBank database are shown. The scale bar represents 0.1 nucleotide substitutions per site. Bootstrap values greater than 70 are indicated at the nodes. Porcine sapelovirus was used as the outgroup

A phylogenetic tree was constructed using 54 nucleotide sequences, comprising 158–162 nucleotides (nt) corresponding to nucleotide positions 217–377 of BEV 5’UTR (Fig. 2). The sequences were from all 54 BEV-positive PCR products amplified with nested RT-PCR, sequenced with detection primer BEVseq-F. Longer 5’UTR nucleotide fragments from 22 selected BEV-positive samples were amplified and sequenced in both directions with primers 41U18 and 611 L21. These sequences, comprising 546–556 nt corresponding to nucleotide positions 61–610 of the BEV 5’UTR, were also analyzed phylogenetically (Fig. 3). Nucleotide numbering was based on the published sequence [GenBank: DQ092794.1]. Variations in the numbers of nucleotides were attributable to insertions or deletions in the region used in the analysis. Compared with published sequences, the phylogenetic trees in both Figs. 2 and 3 suggested that the samples collected in this study contained either EV-E or EV-F. In Fig. 2, the 54 BEV-positive sequences were separated into eight distinct clusters (defined as clusters 1–8) when clades with bootstrap values > 70 % were considered reliable groupings. The eight clusters consisted of three clusters of sequences from group F samples (clusters 1, 4, and 6) and five clusters of sequences from group D and E samples (clusters 2, 3, 5, 7, and 8). The EV-E and EV-F clusters were not reliably separated on the tree in Fig. 2 due to the short length of the sequences analyzed (158–162 nt) and the low bootstrap value (<70 %). However, it was found that the 11 BEV-like sequences from goats (cluster 1) were closely related to the sequence of an uncultured bovine-like enterovirus (Cp3.3) detected in a goat in Spain (bootstrap value > 70 %), with > 86 % sequence identity, and two sequences from cattle (cluster 2) were related to the sequence from the Australian possum (W1), with > 81 % sequence identity.

A subsequent analysis of 22 longer representative 5’UTR sequences (546–556 nt), 14 from cattle, two from gaurs, and six from goats, was performed (Fig. 3). Of the 14 representative BEV sequences from cattle, six clustered with previously reported EV-E sequences and eight with EV-F sequences. The sequence identities were 82–100 % for EV-E and 79–100 % for EV-F sequences. The cattle EV-E sequences were related to the BEV sequences from the Australian cattle (K2577, with > 82 % sequence identity) and the cattle from China (HY12, with > 79 % sequence identity). The cattle EV-F sequences were related to BEV isolated from the United States (Wye 8875, PS87, and BEV-261), with > 86 % sequence identity. Several cattle sequences were related to the sequence from the Australian possum, which was identified as EV-F (W1, with > 87 % sequence identity). Six representatives of BEV-like sequences isolated from goats clustered together, with > 88 % sequence identity. The two sequences from gaurs also clustered together, with > 99 % sequence identity, and were closely related to the EV-F isolate from China (BJ50), with > 94 % sequence identity. When we analyzed the partial 5’UTR sequences of our BEV and BEV-like strains with the published BEV sequences using the Recombination Detection Program (RDP4) [21], no potential recombination was observed in our strains in the genomic region analyzed in this study.

## Discussion

Bovine and bovine-like enteroviruses have been detected in both healthy animals and animals with clinical signs, and also in environmental samples, such as sewage [6, 12]. Although their pathogenicity and virulence is still largely unknown, recent reports have proposed a role for BEV in disease in cattle [16] and experimental animals [20]. BEV have been increasingly isolated from cattle with respiratory disease and diarrhea, suggesting that they can cause disease and should be classified as emerging animal pathogens [15]. The One Health concept, which states that animal health, human health, and environmental health are interconnected, has highlighted zoonotic viral diseases as an issue of significant concern. Enteroviruses are a large group of viruses affecting both humans and animals. A high degree of viral genome recombination has been demonstrated in enteroviruses, both within and between species and serotypes [22–24]. Genome recombination is known to be one factor leading to the emergence of new viruses with altered host range and pathogenicity [25]. Enteroviruses including BEV are also highly stable and can survive for extended periods in both the gastrointestinal tracts of animals and the environment, facilitating their spread [6, 7, 12, 26].

Thailand is an agricultural country with many animal industries, including both open and closed farming systems. Cattle are generally found in rural areas and have extensive human contact. However, to our knowledge, no previous studies have been conducted on the prevalence and diversity of BEV in Thailand. In this study, the high rate at which the BEV and BEV-like 5’UTR was detected and the detection of both EV-E and EV-F species in cattle indicate that BEV are endemic in the study area in Thailand. Genetic variation in the region of 5’UTR was also observed, although no recombination was detected in this genome region. Taken together, the findings of the present study and previous reports from other countries suggest that a study of other genes of the viruses in terms of genetic variation and recombination, their pathogenicity, epidemiology, and animal-animal, animal-environment, and animal-human transmission, are all issues warranting further BEV research.

In this study, the RT-PCR primer pair used for viral detection amplified a region encompassing domains I-IV of the BEV 5’UTR. In the first round of RT-PCR, a band of the expected size was observed in some but not all positive samples. This may be attributable to the small amount of virus in the samples or the low quality of the RNA extracted from the feces, which is known to contain many inhibitors. Nested PCR was used to increase the sensitivity and specificity of detection. The nested PCR primers (BEVseq-F and NBEVseq-R) amplified a region in domains I*-IV, and the amplicon was subjected to nucleotide sequencing. Sequences of 158–162 nt that spanned domains II, III, and IV were retrieved and analyzed. When the nucleotide sequences from all positive isolates were aligned, the nucleotide variability in this region was apparent and was used for phylogenetic analysis (Fig. 2). It has been suggested that in poliovirus, another enterovirus, domain III and the genomic region 5’ to this domain bind polypyrimidine tract-binding protein (PTB), which is a prerequisite for IRES function [27]. Therefore, changes occurring in this genomic region may affect the secondary structure of the 5’UTR, RNA-protein interaction and viral replication, resulting in modifications of cell tropism and pathogenicity of the virus [9, 28]. However, because the amplicon generated with the BEV detection primers was short and a relatively conserved region was used for the analysis, resulting in low bootstrap values (<70 %), we could not classify the sequences into EV-E or EV-F based on the tree in Fig. 2. Therefore, a longer partial nucleotide fragment of the 5’UTR was generated with primers 41U18 and 611 L21. The extended 5’UTR sequences, of 546–556 nt, spanning the region in domains I-V of the BEV 5’UTR, were analyzed phylogenetically and produced high bootstrap values as shown in Fig. 3, indicating that the clustering of the sequences was reliable. This result suggested that the nested RT-PCR using the UniEV-F/BEV-R and BEVseq-F/NBEVseq-R primer pairs which generated PCR products of approximately 300 bp, but < 200 nt of the sequences for phylogenetic study was applicable for the direct detection of BEV in fecal samples, but not for the classification of BEV species. A tentative classification of BEV into EV-E or EV-F based on a phylogenetic analysis of the 5’UTR may be possible when 5’UTR sequences of > 500 nt are used, as shown with some strains in this study. However, according to the current standard, BEV species, serotypes or genotypes are classified on the basis of their capsid genes and polymerase gene, and not on the 5’UTR alone [5]. Therefore, analyses of the capsid genes and polymerase gene are required to confirm the species and serotypes/genotypes of the BEV circulating in Thailand.

Our analysis of the partial BEV 5’UTR sequences from cattle suggests that different BEV variants co-exist among Thai cattle, as has been observed in other regions of the world [6, 12, 29]. The sequence identities observed in this study were 82–100 % for EV-E and 79–100 % for EV-F. It was found in a previous study that other enteroviruses have identities for 70–96 % between isolates [30]. The cattle samples used in this study derived from three herds owned by three separate households. The animals were kept inside during the night but were allowed to roam freely to graze in grassed areas during the day. The phylogenetic tree (Fig. 2) showed a high degree of identity between groups D and E (clusters 2, 3, 5, 7, and 8), whereas group F formed a separate cluster (clusters 1, 4, and 6). As shown in Fig. 1, groups D and E were from nearby households. Unfortunately, the original sources of these animals are unknown. Therefore, no conclusion can be drawn as to whether the strong sequence identity observed in groups D and E are due to a common source of animals or the transmission of BEV among the different cattle herds by environmental contamination with bovine feces.

The sequences of EV-F showed a relatively wide range of identities (79–100 %) and formed at least two clusters on the phylogenetic tree in Fig. 3 (one cattle and one possum cluster), suggesting that these represent true variations among the BEV circulating in the population. Despite minor variations, the 5’UTR region is overall highly conserved, and a sequence identity < 85 % could indicate the presence of BEV variants belonging to different serotypes [5, 12, 15]. Further studies based on structural genes, such as VP1 and VP3, as well as the viral whole genome sequencing, are required to determine whether EV-F in Thailand constitutes a distinct group that is closely related to BEV-like viruses in other animals or is derived from cattle. Notably, the sequences of BEV isolated in this study were related to those found in cattle from China and the United States, and in a possum in Australia, emphasizing the variation in BEV in Thai cattle. This variability also suggests that BEV may be evolving in the cattle host.

In contrast, there was significantly less diversity in the BEV isolated from gaurs and BEV-like viruses from goats, which clustered host specifically and separately from those found in cattle, suggesting host species-specific variations. Previous studies have found that BEV-like viruses from goats are similar to each other but different from those isolated from other animals [6]. BEV-like sequences were highly prevalent (60 %) among goats in Spain in an area with no cattle farming, so goats may not be merely a mechanical carrier of BEV, but a susceptible host [6]. This is further supported by a study in Japan, in which an enterovirus genome was detected in the feces of a goat with diarrhea [14]. Further molecular studies in goats and gaurs are required to determine whether these BEV-like viruses are truly host species-specific or represent new BEV genotypes.

Finally, no BEV-like sequences were detected in any of the deer samples examined. BEV has previously been detected in the feces of the white-tailed deer (*Odocoileus virginianus*) in the United States [12]. However, the deer in our study areas were sambar, hog deer, and Eld’s deer. These deer may be resistant to BEV infection, or may be infected at a prevalence too low for detection in the sample size used here. Alternatively, the 5’UTR of deer enteroviruses may differ sufficiently from those of BEV that the primers used in this study could not detect them.

## Conclusions

Both EV-E and EV-F are endemic viruses in Thai cattle, with different BEV variants circulating in the country. The clustering patterns of sequences in gaurs and goats suggest that these enteroviruses are probably host species-specific. This study is a necessary first step, but more-extensive molecular analyses, particularly of the capsid genes, are required to better understand the biology of these viruses in both domestic and wild animals. As far as we know, this is the first report of the genetic diversity of BEV in Thailand.

## Methods

### Samples

Fecal samples were collected between January and November 2013. They included 60 samples from cattle (*Bos indicus*), 46 samples from domestic goats (*Capra hircus*), 30 samples from gaurs (*Bos gaurus*), and 47 samples from deer, including 23 samples from sambar deer (*Rusa unicolor*), 18 samples from hog deer (*Axis porcinus*), and six samples from Eld’s deer (*Panolia eldii*). All samples were collected from the ground. The animal species were identified by observation of fecal appearance and characteristics by a veterinary specialist. The samples were kept in an ice box (estimated at 0–4 °C) and transferred from Kanchanaburi Province to a laboratory at the Faculty of Tropical Medicine, Bangkok, Thailand, within two days. The samples were then stored at −80 °C until processing.

The collection locations were determined with a Garmin© GPSMap 60CSx set to the Universal Transverse Mercator (UTM) projection (Zone 47P). Satellite images were obtained from LANDSAT (RGB = 742). All geo-referenced data were processed and mapped with ESRI® ArcGIS 9.0.

### Fecal sample processing

A solution of feces from each sample was prepared by diluting the fecal sample with phosphate-buffered saline (PBS) to produce a 30 % (w/v) solution. This solution was mixed by vortexing and was sonicated for 10 min at 4 °C before centrifugation at 1,000 × g for 15 min at 4 °C. The supernatants were collected and stored at −80 °C until the nucleic acid was extracted.

### RNA extraction and BEV 5’UTR RT-PCR

RNA was extracted from 200 μL of fecal solution with the PureLink® Viral RNA/DNA Kit (Invitrogen, Carlsbad, CA), according to the manufacturer’s instructions. Nested RT-PCR was used to detect the BEV 5′UTR in the total RNA extracted from the fecal samples. The first-round one-step RT-PCR was performed with the SuperScript® III One-Step RT-PCR System with Platinum® *Taq* DNA Polymerase (Invitrogen). The RT-PCR reaction contained 1× reaction mix, 0.2 μM forward primer (UniEV-F), 0.2 μM reverse primer (BEV-R), 1 μL of SuperScript® III RT/Platinum® *Taq* Mix and 5 μL of total RNA in a total volume of 25 μL. The RT-PCR cycling parameters were: 50 °C for 30 min for cDNA synthesis, followed by an initial denaturation step at 94 °C for 2 min, 40 cycles of 94 °C for 15 s (denaturation), 50 °C for 30 s (annealing), and 68 °C for 45 s (extension), with a final extension step at 68 °C for 10 min. DreamTaq DNA Polymerase enzyme (Thermo Scientific, Waltham, MA) was used for the second round of the nested PCR. The PCR reaction contained 1× DreamTaq buffer, 0.4 mM dNTPs, 0.2 μM forward primer (BEVseq-F), 0.2 μM reverse primer (NBEVseq-R), 2.5 units of DreamTaq DNA Polymerase, and 1 μL of DNA product from the first-round RT-PCR as the template, in a total volume of 25 μL. The nested PCR cycling parameters were: 98 °C for 2 min as an initial denaturation step, followed by 25 cycles of 98 °C for 30 s (denaturation), 57 °C for 45 s (annealing), and 72 °C for 1 min (extension), with a final extension step at 72 °C for 10 min.

To analyze the extended 5′UTR sequences, a semi-nested RT-PCR was performed on selected BEV-positive samples to amplify a partial nucleotide fragment of the BEV or BEV-like 5′UTR of approximately 600 bp. In the first-round one-step RT-PCR, the same reaction conditions were used as for the BEV 5′UTR detection, with the forward primer 3U23 and the reverse primer 611 L21. The RT-PCR cycling parameters were: 50 °C for 30 min for cDNA synthesis, followed by an initial denaturation step at 94 °C for 5 min, 40 cycles of 94 °C for 1 min, 50 °C for 1 min, and 68 °C for 1 min, with a final extension step at 68 °C for 5 min. The products of the first-round RT-PCR were then subjected to semi-nested PCR using DreamTaq DNA Polymerase and the same reaction conditions used for the BEV 5′UTR detection, with the forward primer 41U18 and the reverse primer 611 L21. The semi-nested PCR cycling parameters were: 98 °C for 2 min, followed by 30 cycles of 98 °C for 30 s, 50 °C for 45 s, and 72 °C for 45 s, with a final extension step at 72 °C for 10 min. The primer sequences are listed in Table 2. All nucleic acid amplifications were performed in an Eppendorf Mastercycler® (Eppendorf, Hamburg, Germany). The PCR products were resolved with 2 % agarose gel electrophoresis, stained with ethidium bromide, and visualized with a UV transilluminator. At the outset of the experiment, no positive control for BEV was available. After the BEV 5′UTR was detected and confirmed with DNA sequencing, a BEV-positive RNA sample was used as the positive control in subsequent RT-PCR.

**Table 2:** Primers for RT-PCR

Primer	Sequence (5′–3′)	Target (positions)^a^	Product size
UniEV-F	GTACCYTTGTRCGCCTGTT	67–85	493 bp
BEV-R	GAGGTTGGGATTAGCAGCATT	539–559	
BEVseq-F	GGGGAGTAGTCCGACTCCGC	124–143	286 bp
NBEVseq-R	CGAGCCCCATCTTCCAGAG	391–409	
3U23	TAAAACAGCCTGGGGGTTGTACC	3–25	629 bp
41U18	CGYGGCGCYAGTACTCTG	41–58	591 bp
611 L21	CCGAAAGTAGTCTGTTCCGCC	611–631	

^a^The nucleotide numbering corresponds to that of the published sequence [GenBank: DQ092794.1]

### Nucleotide sequencing and phylogenetic analysis

For the short 5′UTR PCR fragments (286 bp) generated with the BEV detection primers, the nested PCR products of the expected size were excised from agarose gels, purified, and sequenced directly with the BEVseq-F primer. For the longer 5′UTR fragments (591 bp), the purified products were sequenced directly in both directions with the 41U18 and 611 L21 primers. The PCR products were purified with the GeneJET Gel Extraction Kit (Thermo Scientific) and the products were sequenced by a commercial DNA sequencing company (Macrogen, Seoul, Korea). The nucleotide sequence similarities were determined with the BLAST search algorithm, comparing the sample sequences with those in the National Center for Biotechnology Information (NCBI) GenBank nucleotide database. Sequence contigs derived from two-directional sequencing were joined with CAP (Contig Assembly Program) [31] and the nucleotide sequences were aligned with ClustalW in BioEdit version 7.0.4.1. A phylogenetic tree was constructed in the MEGA 5 program, using the neighbor-joining algorithm with the Kimura two-parameter distance model and 1,000 bootstrap replicates [32]. Sequence identity was determined with the Sequence Identity Matrix function in BioEdit.

### Nucleotide sequence accession numbers

The nucleotide sequences obtained in this study were deposited in the GenBank database. The accession numbers for the sequences from the cattle are KR002738, KR002739, KR002740, KR002741, KR002742, KR002743, KR002744, KR002745, KR002746, KR002747, KR002748, KR002749, KR002750, KR002751, KR002752, KR002755, KR002756, KR002757, KR002758, KR002759, KR002760, KR002761, KR002762, KR002763, KR002764, KR002765, KR002766, KR002767, KR002769, KR002770, KR002771, KR002772, KR002773, KR002774, KR002775, KR002776, KR002777, KR002778, KR002779, KR002780, KT992102, KT992103, KT992104, KT992105, KT992108, KT992109, KT992110, KT992111, KT992112, KT992113, KT992114, KT992115, KT992116, and KT992117. The accession numbers for the sequences from the goats are KR002781, KR002782, KR002783, KR002784, KR002785, KR002786, KR002787, KR002788, KR002789, KR002790, KR002791, KT992118, KT992119, KT992120, KT992121, KT992122, and KT992123. The accession numbers for the sequences from the Indian bison (gaurs) are KR002753, KR002754, KR002768, KT992106, and KT992107.

The accession numbers of the previously published sequences used in the phylogenetic analyses are AF123432.1 (K2577), KF748290.1 (HY12), DQ092769.1 (LC-R4), KM887134.1 (Egypt/5), JQ277724.1 (ovine EV TB4-OEV), AY462106.1 (possum EV W1), AB857843.2 (goat EV G1), AY831718.1 (Cp3.3), HQ917061.1 (BJ50), DQ092794.1 (PS87/Belfast or ATCC VR-774), DQ092770.1 (BEV-261), and AY724745.1 (Wye 8875). JX286666.1 (porcine sapelovirus) was used as the outgroup.
